# ﻿Two new species of *Alainites* (Ephemeroptera, Baetidae) from the Mediterranean biodiversity hotspot

**DOI:** 10.3897/zookeys.1118.84643

**Published:** 2022-08-24

**Authors:** Zohar Yanai, Pavel Sroka, Jean-Luc Gattolliat

**Affiliations:** 1 The Steinhardt Museum of Natural History, Tel Aviv University, Tel Aviv 6997801, Israel Tel Aviv University Tel Aviv Israel; 2 Biology Centre of the Czech Academy of Sciences, Institute of Entomology, Branišovská 31, 37005 České Budějovice, Czech Republic Biology Centre of the Czech Academy of Sciences, Institute of Entomology České Budějovice Czech Republic; 3 Musée cantonal de zoologie, Palais de Rumine, Place de la Riponne 6, 1014 Lausanne, Switzerland Musée cantonal de zoologie, Palais de Rumine Lausanne Switzerland; 4 University of Lausanne (UNIL), Department of Ecology and Evolution, 1015 Lausanne, Switzerland University of Lausanne (UNIL) Lausanne Switzerland

**Keywords:** COI, Israel, Italy, mayfly, microendemics, Sardinia, systematics, West Palaearctic

## Abstract

The mayfly genus *Alainites* Waltz & McCafferty, 1994 encompassed 20 species and was represented across the West Palaearctic region by six species. Based on morphological (nymphal characters) and molecular (mitochondrial COI sequences) evidence, two new species are described: *A.bengunn***sp. nov.** from Sardinia and *A.gasithi***sp. nov.** from Israel. Both species are confined to narrow distribution ranges, in line with most of their congeners from the region. The key nymphal traits are discussed and identified to distinguish species in the group.

## ﻿Introduction

*Baetis* Leach, 1815 (Ephemeroptera: Baetidae) is one of the most diversified mayfly genera (Sroka 2012). In order to better understand its phylogenetic structure, as well as for practical reasons, attempts are made to divide it into monophyletic taxa, ranked as either species groups, subgenera, or independent genera, siblings to *Baetis*. One of the first to suggest such a division for the European fauna was Müller-Liebenau (1969), who divided *Baetis* s.l. into eleven species groups. The concept of *Baetis* s. l. was subsequently recognised as polyphyletic, especially when considering taxa from other biogeographic regions (e.g., Novikova and Kluge 1987; Waltz et al. 1994; Waltz and McCafferty 1997; Fujitani 2008; Gattolliat et al. 2008). Most of the species groups were proven to correspond to independent lineages and part of them were raised to subgeneric or generic levels (Novikova and Kluge 1987; Waltz et al. 1994; McCafferty and Waltz 1995).

*Nigrobaetis* Novikova & Kluge, 1987 and *Alainites* Waltz & McCafferty, 1994 were erected for species mainly belonging to the species groups *niger* and *muticus*, respectively, sensu Müller-Liebenau (1974). *Takobia* Novikova & Kluge, 1987 was established for a single species originally described from Uzbekistan by Braasch and Soldán (1983) under the binomial combination “*Centroptilummaxillare*”. The status of these three taxa was rather hectic as they were repeatedly subject to synonymies and rank changes between species groups, subgenera, and genera (Müller-Liebenau 1969; Novikova and Kluge 1987, 1994; Waltz et al. 1994; Waltz and McCafferty 1997; Jacob 2003; Kluge and Novikova 2014). This confusion is partly due to the fact that some of these acts lack solid morphological or molecular support; moreover, the revisions were not based on type material and detailed descriptions were missing (see Sroka et al. 2021).

When Waltz and McCafferty (in Waltz et al. 1994) established the genus *Alainites*, the *muticus* group sensu Müller-Liebenau (1974) included nine species; all of them were reassigned to the new genus (Waltz et al. 1994). Diagnostic nymphal characters of *Alainites* were: 1) paraproct with an elongated prolongation; 2) prostheca of the right mandible bifid, reduced to two bristle-like feathered appendages; 3) absence of villopore; and 4) body laterally compressed. At the imaginal stage: 1) hindwings, when present, with three longitudinal veins, the second being bifurcated; and 2) segment III of the male forceps spherical to slightly elongated and curved (Waltz et al. 1994; Zrelli et al. 2012). It is worth mentioning that none of the imaginal characters allows the separation of *Alainites* from its allied genus *Nigrobaetis* (Novikova & Kluge 2014). The *Alainites* concept was widely debated in the literature (Tong and Dudgeon 2000; Fujitani et al. 2003; Gattolliat 2011; Zrelli et al. 2012). In some studies focusing on European fauna, *Alainites* was still tentatively considered a synonym of *Baetis* s.l. (Jacob 2003) or *Nigrobaetis* (Bauernfeind and Soldán 2012). Kluge and Novikova (2014) considered *Alainites* as a junior synonym of *Takobia* Novikova & Kluge, 1987, and transferred de facto all the species of *Alainites* into *Takobia*. They justified this synonymy by a strict application of the principles of phylogenetic systematics (Hennig 1950), as they considered *Takobiamaxillare* (Braasch & Soldán, 1983) as highly derived within the lineage and the remaining species previously assigned to *Alainites* as plesiomorphic. Therefore, to avoid keeping *Alainites* as a plesiomorphon, they synonymised it with *Takobia*. However, for a long time *T.maxillare* remained, as Kluge and Novikova 2014 wrote, a “single aberrant species”. Recently Sroka et al. (2021) provided a redescription of the type species of *Takobia* based on the type material; important characters, such as the prosthecas and the paraprocts, were re-examined and corrected. Moreover, two new species were described form Central Asia which presented derived characters similar to *T.maxillare*, namely a very elongated maxillary palp, the dorsal surface of the labrum covered with numerous setae (none of them arranged in a row), the peculiar setation of the dorsal margin of femora, and elongated claws.

Currently *Alainites* is widely distributed across the Palaearctic (represented by 13 species) as well as in the Oriental realm (7 species). In Oriental realm, *Alainites* is reported both from continental areas (mainly China and Malaysia) and from a few islands (Taiwan and Borneo). Ongoing studies in Thailand, Philippines, and Indonesia clearly indicate that the genus is more widely distributed in the region, but seems nowhere very common (J. Garces, C. Suttinun, pers. comm.). During the study of other Baetidae from South East Asia, it was never found eastern to Wallace Line (i.e. in New Guinea and nearby islands) (Kaltenbach and Gattolliat 2017, 2018, 2019a, 2019b). Unlike its closely related genus *Nigrobaetis*, *Alainites* is absent from Afrotropics including the Arabian Peninsula.

In Western Palaearctic, *Alainitesmuticus* (Linneaus, 1758) is the most common and widely distributed species of the genus. It has been reported across Europe, from Portugal to Ukraine and from Greece to Scandinavia (Bauernfeind and Soldán 2012), with highest densities exhibited in Central Europe, where it is often one of the most common mayfly species. It was also recently reported from Armenia (Hrivniak et al. 2018) and Iran (Bojková et al. 2018). Based on molecular evidence, *A.muticus* seems to represent in fact a complex of at least two cryptic species (Sroka 2012); however, no morphological studies have validated these species hypotheses to date.

Just behind the limit of distribution of *A.muticus*, species with restricted distribution were described over the last three decades. Sympatry amongst West Palaearctic *Alainites* species has very seldomly been recorded and is probably very rare, given the restricted distribution range of most species. In the Maghreb, *Alainitesoukaimeden* (Thomas & Sartori, 1992) occurs in the High Atlas (Morocco), whilst *Alainitessadati* Thomas, 1994 is found from West Algeria to North Tunisia (Thomas and Gagneur 1994; Zrelli et al. 2012). The westernmost species is *A.navasi* (Müller-Liebenau, 1974), known from the Iberian Peninsula (Müller-Liebenau 1974). In the eastern border of the distribution of *A.muticus*, *Alainiteskars* (Thomas & Kazancı, 1989) was described from Turkey (Kazancı and Thomas 1989), and Nigrobaetis (Takobia) katerynae Martynov & Godunko, 2017 was recently discovered from the Caucasus (Martynov and Godunko 2017); the latter species was never formally transferred to *Alainites*.

In Corsica, an endemic species, *Alainitesalbinatii* (Sartori & Thomas, 1989) was described based on nymphs and imagos (Sartori and Thomas 1989). In the sister island of Sardinia, a population of *Alainites* was firstly considered to be the continental *A.muticus* (Buffagni et al. 2003). In the barcoding of the Italian mayflies project, the Sardinian lineage was tentatively considered as A.sp.cf.albinatii without further explanations (Tenchini et al. 2018). However, morphological and molecular approaches proved that Sardinian specimens of *Alainites* noticeably differed from both the Corsican endemic and the continental lineages (Gattolliat et al. 2015; Tenchini et al. 2018). Gattolliat et al. (2015) noticed that the number of abdominal gills and the structure of the prolongation of the paraproct significantly differed between the Sardinian, Corsican and continental specimens; they even proposed that *Alainites* could be the only lineage present on both Corsica and Sardinia which demonstrates more affinities between Sardinia and Maghreb than between the two islands. The Sardinian lineage was therefore considered as a species hypothesis without formal description (Gattolliat et al. 2015).

For the Levant area, Koch (1988) did not mention any species which could be assigned to *Alainites*. He only reported for the first time two species of the related genus *Nigrobaetis* (*N.niger* and *N.digitatus*) from Syria. In his unpublished master thesis, Samocha (1972) mentioned the presence in Israel of one unidentified species with some probability to be a representative of *Alainites*. The species L55 is characterised by having six pairs of gills and bifid, thin right prostheca (Samocha 1972).

In the current paper, we describe two species of *Alainites* that join the six species of *Alainites* distributed in the circum-Mediterranean region. The Mediterranean basin is recognised as a biodiversity hotspot (Myers et al. 2000). Mediterranean stream biota is unique and featuring a high rate of endemic species (Bonada et al. 2007), including over one third of its mayfly species (Tierno de Figueroa et al. 2013). Stream macroinvertebrates exhibit a clear set of traits that make them suitable for the typical Mediterranean climatic and hydrological conditions (Hershkovitz and Gasith 2013). The combination of specialised fauna, unique environment, and heavy anthropogenic pressure results in a constant threat to these fragile aquatic insects (Filipe et al. 2012).

## ﻿Materials and methods

The material treated here includes nymphs, that have been collected using a hand net or picked manually from rocks and pebbles. All material is preserved in ethanol, except for a few specimens that have been mounted on microscope slides fixed in Canada Balsam, as specified in the material examined sections below. Ethanol-preserved specimens were studied under a Leica M205 stereomicroscope; microscope slides were drawn from a drawing tube mounted on an Olympus BX51 compound microscope. Material is deposited in the Musée Cantonal de Zoologie at Lausanne, Switzerland (**MZL**), Steinhardt Museum of Natural History at Tel Aviv University, Israel (**SMNH**), and Biology Centre of the Czech Academy of Sciences, Institute of Entomology (**IECA**).

DNA for species delineation was extracted using the non-destructive protocol outlined by Vuataz et al. (2011), which enabled post-extraction morphological study of specimens. The ‘barcoding section’ of the mitochondrial cytochrome *c* oxidase subunit I (**COI**) was PCR-amplified with the primers HCO2198 and LCO1490 (Folmer et al. 1994). Amplification followed the conditions and protocols outlined by Sroka et al. (2021). Automated sequencing was carried out in Microsynth (Balgach, Switzerland).

Molecular reconstruction was conducted on the four newly obtained sequences from Israel and three already published sequences from Sardinia (Suppl. material 1). Forty-six additional reference sequences of representative Palaearctic *Alainites* species and allied genera were obtained from GenBank (http://www.ncbi.nlm.nih.gov/), from the unpublished FREDIE database (http://wp.fredie.eu//), or sequenced for the first time (an individual of *A.sadati* from Algeria). GenBank accession numbers are available in Suppl. material 1. Sequence chromatograms were inspected and edited using Geneious v. 7.1.5 (Biomatters Ltd.). Alignment, reconstruction, and genetic distance calculations were conducted in MEGA-X v. 10.0.5 (Kumar et al. 2018). A maximum likelihood (**ML**) analysis was conducted in RAxML v. 8 (Stamatakis 2014; implemented in raxmlGUI v. 2.0.7, Edler et al. 2021), employing HKY+I as sequence evolution model, with 100 runs and 1000 bootstrap replicates.

## ﻿Results

In comparison to COI sequences of other available taxa in *Alainites* and allied genera, the two newly described species demonstrate very low intraspecific variation (up to 0.7%) and very high interspecific distances (at least 19.2%).

### ﻿ Alainites bengunn

Taxon classificationAnimaliaEphemeropteraBaetidae

Yanai & Gattolliat sp. nov.

A3165CB4-692D-582E-BB02-7DC02A2067BA

https://zoobank.org/A4EA23BC-7DEE-4587-88E7-D27BCA64EE31

Figs 1 , 2 , 3

Alainites muticus in Buffagani et al. 2003
Alainites cf. muticus in Gattolliat et al. 2015
Takobia sp. cf. albinatii in Tenchini et al. 2018

#### Material examined.

***Holotype*.** 1 nymph, Italy, Sardinia, Loc near Fonni, trib. of Taloro (SA27), 40°09.05'N, 9°16.37'E, alt. 810 m a.s.l., 15.v.2009, L. Vuataz, E. Cavallo & Y. Chittaro leg., MZL (GBIFCH 00970536). ***Paratypes*.** Italy. 3 nymphs (2 on slide), same details as holotype, SMNH, IECA, MZL (GBIFCH 00280204, GBIFCH 00604446, GBIFCH 00604447) • 21 nymphs (2 on slide), Sardinia, Monti del Genargentu, near Monte Spada, 20.vi.2010, T. Soldán leg., MZL (7 nymphs), SMNH (7 nymphs), IECA (7 nymphs) • 33 nymphs (2 on slide), Sardinia, tributary of Fiume Taloro Riv., Fonni village, 20.vi.2010, T. Soldán leg., MZL (11 nymphs), SMNH (11 nymphs), IECA (11 nymphs) • 35 nymphs (2 on slide), Sardinia, Riu Pramaera River, Lotzorai village, 21.vi.2010, T. Soldán leg., MZL (11 nymphs), SMNH (11 nymphs), IECA (13 nymphs). **Additional non-type material.** Italy. 3 nymphs, Sardinia, Rio Rumine, vicinity of Mamoiada village, 20.vi.2010, T. Soldán leg., IECA • 1 nymph, Sardinia, Genna Stream, Auxi Pass, vicinity of Urzulei village, 21.vi.2010, T. Soldán leg., IECA.

#### Material not examined.

Italy. Sardinia, Loc. Siligo village, 40°35.36'N, 8°43.55'E, alt. 240 m a.s.l., C. Belfiore leg.

#### Differential diagnosis.

The species is distinct amongst other West Palaearctic *Alainites* species based on the combination of (1) six pairs of abdominal gills, (2) paraproct prolongation covered with spines on its entire surface, (3) serration between prostheca and mola, and (4) low number of dorsal setae on its fore-femora (14–20) and relatively many of them on the fore-tibiae (up to 17).

#### Description of nymph.

***Length***. Female body 7.0–7.9 mm; cerci 4.5–5.5 mm; median caudal filament ca. 2/3 of cerci. Male body 6.0–6.7 mm; cerci 4.0–5.0 mm; median caudal filament ca. 2/3 of cerci.

**Figure 1.**
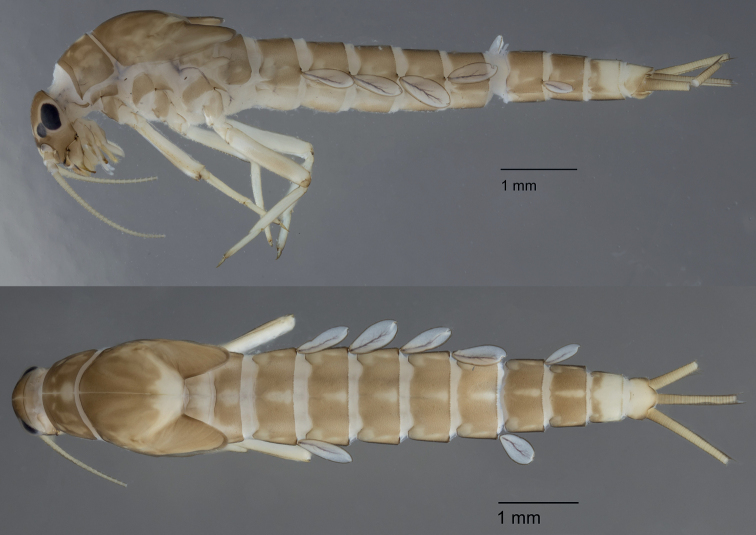
*Alainitesbengunn* sp. nov., habitus: lateral view (top) and dorsal view (bottom).

***Colouration*** (Fig. 1). General colouration brown. Head uniformly brown with vermiform marks on vertex and frons. Legs ecru except upper side of femora brown. Thorax brown with some paler area, but without clear pattern. Abdominal tergites brown with a central yellowish elongated dot; distal part of tergite IX and whole tergite X yellowish. Abdominal sternites I and II yellowish, III–IX pale brown. Cerci ecru to pale brown.

**Figure 2.**
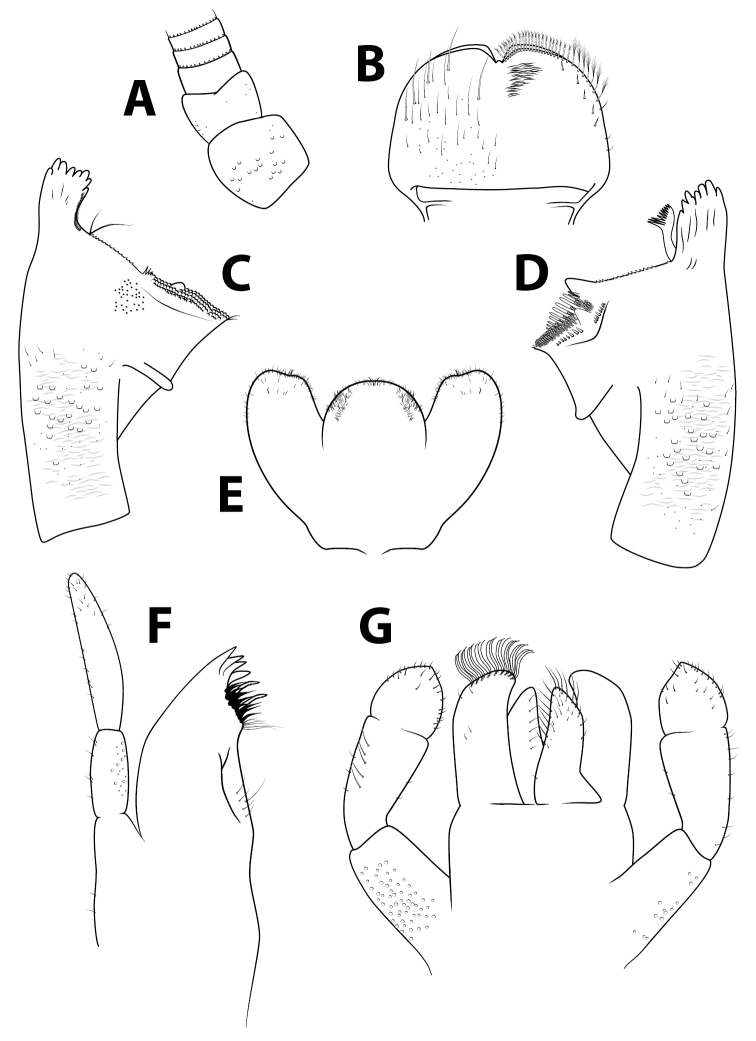
*Alainitesbengunn* sp. nov., nymph, characters of the head **A** antenna base **B** labrum (left side dorsal view, right side ventral view) **C** right mandible (ventral view) **D** left mandible (ventral view) **E** hypopharynx **F** maxilla **G** labium (left side dorsal view, right side ventral view). **B–G** presented to the same scale.

***Head*.** Antennae (Fig. 2A) close to each other, with a narrow interantennal carina; scape with few deep scale insertions. Dorsal surface of labrum (Fig. 2B) with one central long seta and distolateral arc of four medium to long, simple, stout setae, and small fine setae scattered on surface; ventral surface with 5–6 submarginal small, pointed setae; distal margin fringed with ca. 20 short, followed by seven or eight long, feathered setae. Right mandible (Fig. 2C) slightly shagreened, with sparse fine setae and deep scale insertions; incisors composed of eight apically rounded, distinct denticles, outer- and innermost denticles smaller than others; prostheca reduced and bifid with numerous thin setae; space between prostheca and mola serrated, tuft of setae absent; apex of mola with tuft of two setae. Left mandible (Fig. 2D) slightly shagreened, with sparse fine setae and deep scale insertions; incisors composed of eight rounded, distinct denticles, outer- and innermost denticles smallest, and small denticle in the middle; prostheca with few medium denticles and comb-shaped structure; margin between prostheca and mola serrated, without setae; tuft on apex of mola reduced to one seta. Hypopharynx (Fig. 2E) trilobed, apically sparsely covered with thin setae; lingua with no central protuberance; superlingua subequal to lingua. Maxillae (Fig. 2F) apex with three elongated and curved teeth and a tooth-like dentiseta; crown with two rows of setae, first row with small setae, second row with two long stout feathered dentisetae; palp two-segmented, extending the apex of galealacinia, length of segment I approximately 0.75 × segment II; segment II apically rounded, with sparse thin setae. Labium (Fig. 2G) with glossae slightly shorter than paraglossae; inner margins of glossae with eight or nine stout medium setae, apical margin with ca. nine long stout setae; ventral surface with few thin scattered setae; dorsal surface with row of ca. six medium setae; paraglossae with three rows of eight or nine long, stout, simple setae apically; labial palp three-segmented; segment I 0.75 × length of segments II and III combined; segment II with dorsal oblique row of five medium setae; segment III rounded, nearly symmetrical, slightly pointed apically, covered with few short thin setae.

**Figure 3.**
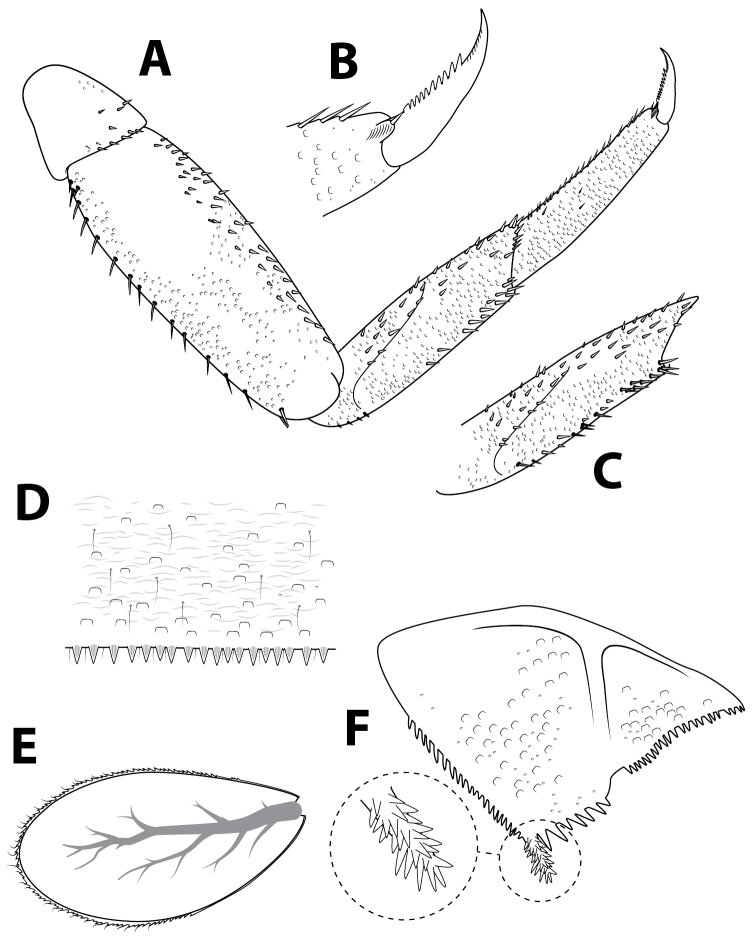
*Alainitesbengunn* sp. nov., nymph, characters of the thorax and abdomen **A** foreleg **B** tarsal claw of foreleg **C** tibia of midleg **D** distal margin of tergum IV **E** gill IV **F** paraproct (with detail of prolongation).

***Thorax*.** Forelegs (Fig. 3A). Trochanter with four or five marginal short stout pointed setae and few similar setae on surface. Femora dorsally with one row of 14–20 long, stout setae, and very few setae subparallel to dorsal margin; dorsoapical setal patch formed by two stout, long setae; ventrally several marginal and submarginal short stout pointed setae; lateral surface with scale bases, mainly on apical half and along subdorsal area. Tibiae dorsally with 9–17 short stout pointed setae, denser towards apical end, few proximal minute setae; ventrally with marginal and submarginal short stout pointed setae, denser towards apical end; tibiopatellar suture present; lateral surface with few short, stout, pointed setae and numerous scale bases. Tarsi bare dorsally; ventral margin with ca. 20 pointed medium setae; lateral surface with numerous scale bases. Tarsal claws (Fig. 3B) hooked with one row of 10–15 medium teeth, apical setae absent. Mid and hindlegs similar to forelegs except femora dorsally with 13–25 pointed setae and tibiae (Fig. 3C) with 11–18 similar setae dorsally. Hindwing pads present.

***Abdomen*.** Terga (Fig. 3D) shagreened, with numerous scale bases, distal margin of tergite IV with triangular spines about twice longer than broad. Sterna shagreened with scales and scale bases; posterior margin smooth without spination. Gills (Fig. 3E) on segments II–VII, elliptical, almost symmetrical and with serrated margins, except proximal part; tracheation well visible and well branched; gill VII similar to gills II to VI. Paraproct (Fig. 3F) with abundant scale bases but almost no setae; margin with ca. seven broad, triangular spines inner to prolongation and numerous medium spines outer to prolongation; prolongation covered with numerous small spines; cercotractor with scattered scale bases, margin with ca. 20 small spines.

#### Imagos.

Unknown.

#### Etymology.

The species is endemic to an island, just like Ben Gunn who was deserted and isolated on an island by his crewmates in “Treasure Island” by R.L. Stevenson (1850–1894).

#### Distribution and ecology.

Little is known about the ecology and distribution of this species. The type locality is a small stream (less than three meters wide), shallow, and with medium current velocity. The species is only known from Sardinia where it is apparently not frequent and not very abundant.

### ﻿ Alainites gasithi

Taxon classificationAnimaliaEphemeropteraBaetidae

Yanai & Gattolliat sp. nov.

6D652198-674D-51D6-BB44-279ECA66E611

https://zoobank.org/6445DE52-25A3-49AF-92A4-FCE665C57753

Figs 4 , 5 , 6

species L55 in Samocha 1972

#### Material examined.

***Holotype*.** Israel. 1 female nymph; Wadi Al-Qassab, Maymon Spring, 33°06.74'N, 35°39.62'E, 290 m a.s.l., 4.iv.2016, Z. Yanai leg., SMNH (385900). ***Paratypes*.** Israel. 9 nymphs, same data as holotype. 5 nymphs SMNH (385901), 2 nymphs MZL (GBIFCH 00972062), 2 nymphs IECA • 10 nymphs, Maymon Spring, 33°06.74'N, 35°39.62'E, 290 m a.s.l., 22.vi.2014, Z. Yanai leg., 7 nymphs SMNH (385895), 3 nymphs MZL (GBIFCH 00971882) • 2 nymphs, same locality, 13.iv.2018, Z. Yanai leg., SMNH (385896) • 4 nymphs (1 on slide), same locality, 26.iii.2019, Z. Yanai leg., SMNH (385892, 385893, 385894) • 10 nymphs (2 on slides), Tina (Nutra) Stream, 33°04.70'N, 35°38.63'E, 72 m a.s.l., 15.vii.2014, Z. Yanai leg., SMNH (385902, 385903, 385904, 385905) • 5 nymphs, same locality, 6.xi.2015, Z. Yanai & S. Cohen leg., SMNH (385898, 385899) • 1 nymph, same locality, 16.v.2016, Z. Yanai & A. Charvet leg., SMNH (385897) • 1 nymph, same locality, 10.iii.2017, Z. Yanai & J.-L. Gattolliat leg., MZL (GBIFCH 00971972) • 4 nymphs (1 on slide), same locality, 27.iii.2019, Z. Yanai leg., SMNH (385906, 385907) • 7 nymphs (1 on slides), Gilbon Stream, old mill, 33°02.45'N, 35°38.40'E, 76 m a.s.l., 29.x.2015, Z. Yanai leg., SMNH (385889, 385890) • 4 nymphs, Divsha Stream, 33°05.41'N, 35°38.90'E, 150 m a.s.l., 6.xi.2015, Z. Yanai & S. Cohen leg., SMNH (385887, 385888) • 1 nymph, ‘Ayit Waterfall, 32°57.28'N, 35°45.23'E, 470 m a.s.l., 4.iv.2016, Z. Yanai leg., SMNH (385891). **Additional non-type material.** Israel. 1 nymph, Jordan River, ‘Ateret Fortress, 33°00.19'N, 35°37.72'E, 63 m a.s.l., 16.v.2016, Z. Yanai & A. Charvet leg., SMNH (385908).

#### Differential diagnosis.

The species is distinct amongst other West Palaearctic *Alainites* species based on the combination of (1) six pairs of abdominal gills, (2) paraproct prolongation with spines only along the border, (3) serration between prostheca and mola, and (4) low number of dorsal setae on its fore-femora (10–20) and fore-tibiae (6–12).

#### Description of nymph.

***Length*.** Female body 3.7–4.0 mm; cerci broken; median caudal filament 1.3–1.4 mm (ca. 2/3 of cerci); male body 3.7–3.9 mm; cerci broken; median caudal filament ca. 2/3 of cerci.

**Figure 4.**
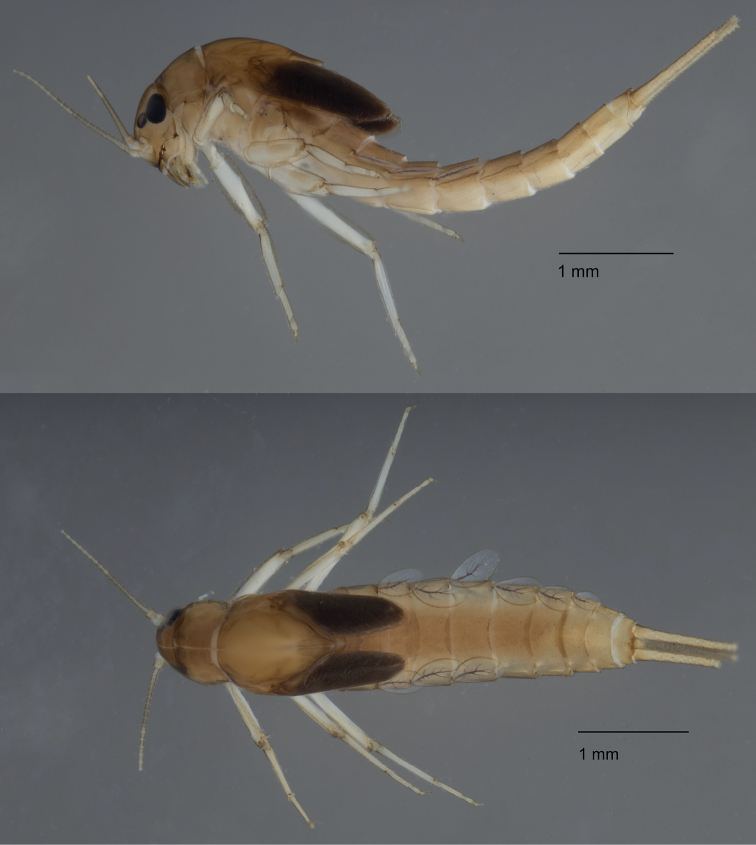
*Alainitesgasithi* sp. nov., habitus: lateral view (top) and dorsal view (bottom).

***Colouration*** (Fig. 4). General colouration pale to medium brown. Head uniformly pale brown with vermiform marks faintly visible on vertex and frons. Turbinate eyes in male nymphs medium brown. Legs ecru, except a broad area on upper side of femora. Thorax medium brown without mark or pattern. Abdominal tergites medium brown without any pattern. Abdominal sternites pale to medium brown. Cerci ecru to pale brown without bands or pattern.

**Figure 5.**
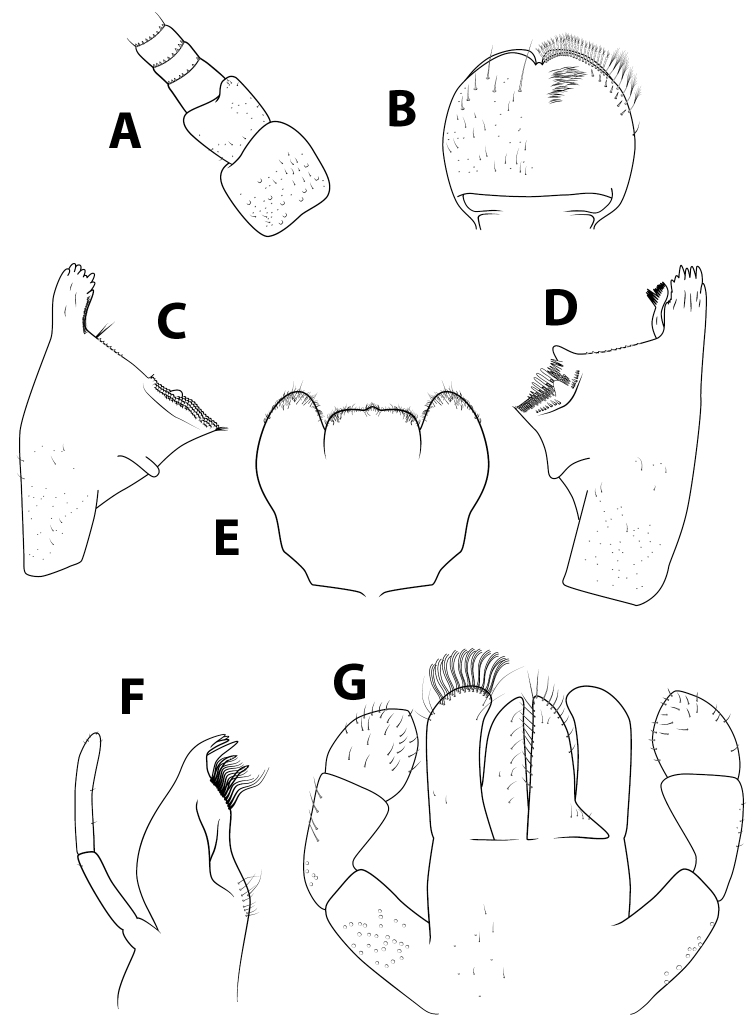
*Alainitesgasithi* sp. nov., nymph, characters of the head **A** antenna base **B** labrum (left side dorsal view, right side ventral view) **C** right mandible (ventral view) **D** left mandible (ventral view) **E** hypopharynx **F** maxilla **G** labium (left side dorsal view, right side ventral view). **B–G** presented to the same scale.

***Head*.** Antennae (Fig. 5A) close to each other, with a narrow interantennal carina; scape with few deep scale insertions and few setae. Dorsal surface of labrum (Fig. 5B) with one central long seta, distolateral arc of three or four simple medium to long, stout setae, and scattered small fine setae; ventral surface with 5–10 submarginal small pointed setae; distal margin fringed with ca. 20 short, followed by seven or eight long, feathered setae. Right mandible (Fig. 5C) smooth, not shagreened, with sparse fine setae; incisors composed of eight apically rounded, distinct denticles, outer- and innermost denticles smaller than others; prostheca reduced and bifid with numerous thin setae; outer half of margin between prostheca and mola serrated, tuft of setae absent; apex of mola with tuft of setae. Left mandible (Fig. 5D) smooth, with sparse fine setae; incisors composed of seven apically rounded, distinct denticles, outer- and innermost denticles smallest; prostheca with medium denticles and comb-shaped structure; margin between prostheca and mola almost entirely serrated, without setae; apex of mola with tuft of setae. Hypopharynx (Fig. 5E) trilobed, apically covered with thin setae; lingua with small central protuberance; superlingua slightly longer than lingua. Maxillae (Fig. 5F) apex with three elongated and curved teeth and a tooth-like dentiseta; crown with two rows of setae, first row with small setae, second row with two long stout feathered dentisetae; palp two-segmented, reaching or slightly exceeding the apex of galealacinia, length of segment I subequal to segment II; segment II apically rounded, with few thin setae. Labium (Fig. 5G) with glossae subequal to paraglossae; inner margins of glossae with 7–10 stout medium setae, apical margin with 7–11 long stout setae, ventral surface with few thin scattered setae; dorsal surface with row of ca. 7 medium setae; paraglossae of constant width, with three rows of 10–12 long, stout, simple setae apically; labial palp three-segmented; segment I nearly half the length of segments II and III combined; segment II with dorsal oblique row of four medium setae; segment III conical, asymmetrical.

**Figure 6.**
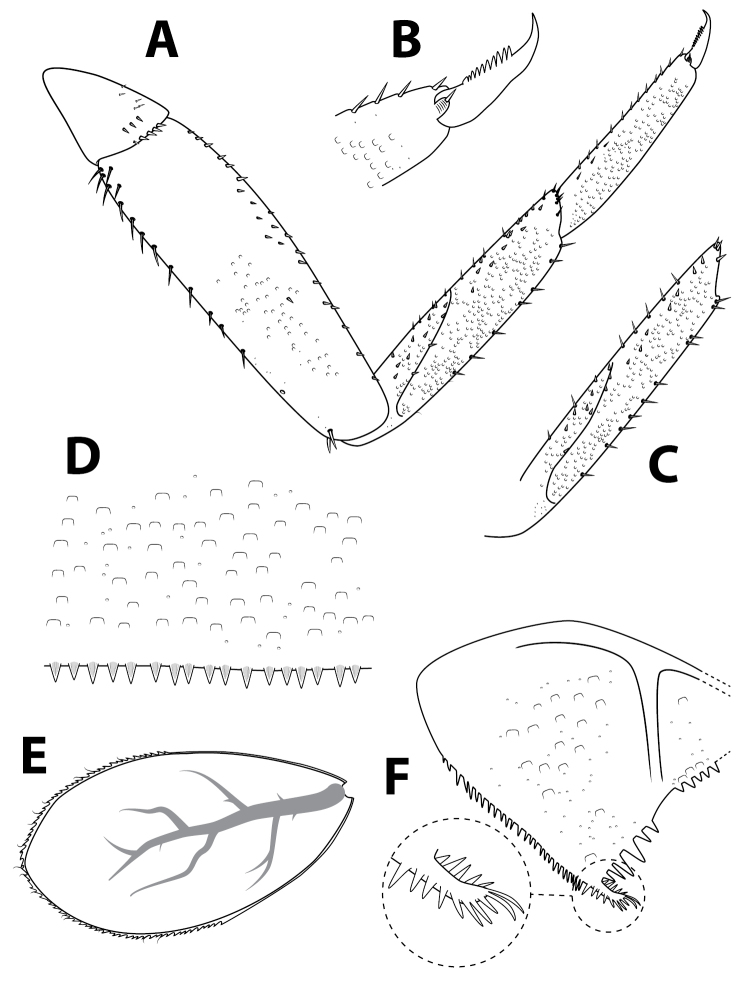
*Alainitesgasithi* sp. nov., nymph, characters of the thorax and abdomen **A** foreleg **B** tarsal claw of foreleg **C** tibia of midleg **D** distal margin of tergum IV **E** gill IV **F** paraproct (with detail of prolongation).

***Thorax*.** Forelegs (Fig. 6A). Trochanter with four or five marginal short stout pointed setae. Femora dorsally with one row of 10–20 medium, stout setae; dorsoapical setal patch formed by two stout, medium setae; ventral margin with pointed short setae; lateral margin with sparse scale bases, mainly on apical half. Tibiae dorsally with ca. six (rarely up to 10–12) setae and single apical seta; ventral margin with small pointed scales and apical patch formed of four or five stout setae; tibiopatellar suture present; lateral margins with few scales and numerous scale bases. Tarsi bare dorsally; ventral margin with 10–15 small pointed setae; lateral margins with numerous scale bases. Tarsal claws (Fig. 6B) hooked with one row of 7–13 (usually 10–11) medium teeth, apical setae absent. Mid and hindlegs similar to forelegs, except midtibiae (Fig. 6C) usually with 9–11 pointed setae on the dorsal margin and hind tibiae usually with 5–7 such setae. Hindwing pads present.

***Abdomen*.** Terga (Fig. 6D) with numerous scale bases, smooth, not shagreened, distal margin of tergite IV with triangular spines, at least twice longer than wide. Sterna shagreened with scales and scale bases; posterior margin smooth without spination. Gills (Fig. 6E) on segments II–VII, elliptic, symmetrical and serrated on margins of distal half; tracheation well visible and well divided; gill VII similar to gills II to VI. Paraproct (Fig. 6F) with abundant scale bases, no setae; margin with six or seven triangular spines of varying size inner to prolongation and numerous medium spines outer to prolongation; prolongation margined with about 15 elongated medium spines, without spines on ventral surface; cercotractor with scale bases, margin with medium spines.

#### Imagos.

Unknown.

#### Etymology.

The name is a noun in apposition. The first author dedicates the species to his former mentor Prof. Avital Gasith (1943–). He is, in many aspects, the founder of freshwater ecology research in Israel. He trained the majority of the local active experts and contributed significantly to our understanding of freshwater systems and taxa, and to their conservation.

#### Distribution and ecology.

Typical habitats of *A.gasithi* sp. nov. include spring-fed brooks in the western slopes of the Golan Heights, with shallow running waters upon basalt bedding. Little is known about the seasonality of this species as it has been rarely collected. Mature nymphs were collected in the spring and early summer (late March to June). Interestingly, the examined specimens were collected mainly in 2014–2019, and despite continuing research and much effort in the same sites, the species was not collected in 2020–2021, an observation that may indicate inter-annual fluctuations in population sizes. However, the species is very rare even in positive sampling event, suggesting that further research is needed for estimation of meta-population structure and stability.

## ﻿Discussion

### ﻿Morphological characters of *A.bengunn* sp. nov. and *A.gasithi* sp. nov.

The two newly described species are assigned to *Alainites* since they share all the synapomorphic characters of the genus, especially laterally compressed body, elongated paraproct prolongations, and prostheca of the right mandible composed of two feathered bristles (Waltz et al. 1994). Within *Alainites*, the nymphs of the two species are distinct from the most common species *A.muticus* by the number of gills (*A.muticus* is the only West Palearctic species of *Alainites* with seven pairs). They differ from other West Palaearctic congeners as follows (see also Table 1):

**Table 1.** Diagnostic characters of West Palaearctic *Alainites* species.

Species	Distribution	Left mandible: margin between prostheca and mola	Mandible lateral side	Fore-femur dorsal margin: number of spines	Fore-tibia dorsal margin: number of spines	Number of gill pairs	Cuticle abdominal terga and sterna	Tergite IV: spines on distal margin	Prolongation of paraproct
*Alainitesalbinatii* (Sartori & Thomas, 1989)	Corsica	10 small teeth	scale bases, shagreened	15	6	6	slightly shagreened	long triangular, pointed	apically covered by spines
*Alainitesbengunn* sp. nov.	Sardinia	serrated	scale bases, slightly shagreened	14–20	9–17	6	shagreened	slightly lanceolate	covered by spines
*Alainitesgasithi* sp. nov.	Israel	serrated	no scale bases, almost not shagreened	10–20	ca. 6, rarely 10–12	6	smooth	long triangular, pointed	spines only on border
*Alainiteskars* (Thomas & Kazancı, 1989)	Turkey	only minute serration close to mola	no scale bases, almost not shagreened	> 40 in two rows	5–9	6	slightly shagreened	triangular, pointed	spines on entire surface (Martynov and Godunko 2017) or just on apex (Kazancı and Thomas 1989)
*Alainitesmuticus* (Linnaeus, 1758)	Palaearctic	10 small teeth	rare scale bases	14	8	7	slightly shagreened	short triangular, broad basally	spines only on border
*Alainitesnavasi* (Müller-Liebenau, 1974)	Iberian Peninsula	10 small teeth	?	26	21	6	smooth	short triangular	covered by spines
*Alainitesoukaimeden* (Thomas & Sartori, 1992)	Morocco (High Atlas)	10 small teeth	shagreened	20	8	6	strongly shagreened	long, relatively narrow	covered by spines
*Alainitessadati* Thomas, 1994	Algeria, Tunisia	10 small teeth	no scale bases, almost not shagreened	ca. 25	6–9	6	slightly shagreened	medium triangular	covered by spines

*Alainitesbengunn* sp. nov. can be distinguished from *A.gasithi* sp. nov. based on a presence of spines on all the surface of the paraproct prolongation (spines only on margins in *A.gasithi* sp. nov.). Spines restricted to the prolongation margin were originally also reported for *A.kars* by Kazancı and Thomas (1989: fig. 7). It is worth mentioning, that later Martynov and Godunko (2017: figs 45, 46) depicted specimens of *A.kars* from Armenia with spines on all the surface of paraproct prolongation. Additionally, *A.kars* possesses many more setae on the dorsal margin of femora (more than 40 on the forefemur of *A.kars* according to Kazancı and Thomas 1989), and lacks the serration between the prostheca and mola. From *A.navasi*, occurring in the West Mediterranean, *A.bengunn* sp. nov. can be distinguished by the more shagreened surface of terga, together with fewer dorsal setae on femora and tibiae (ca. 26 and ca. 21 in *A.navasi*, respectively, according to Zrelli et al. 2012).

**Figure 7.**
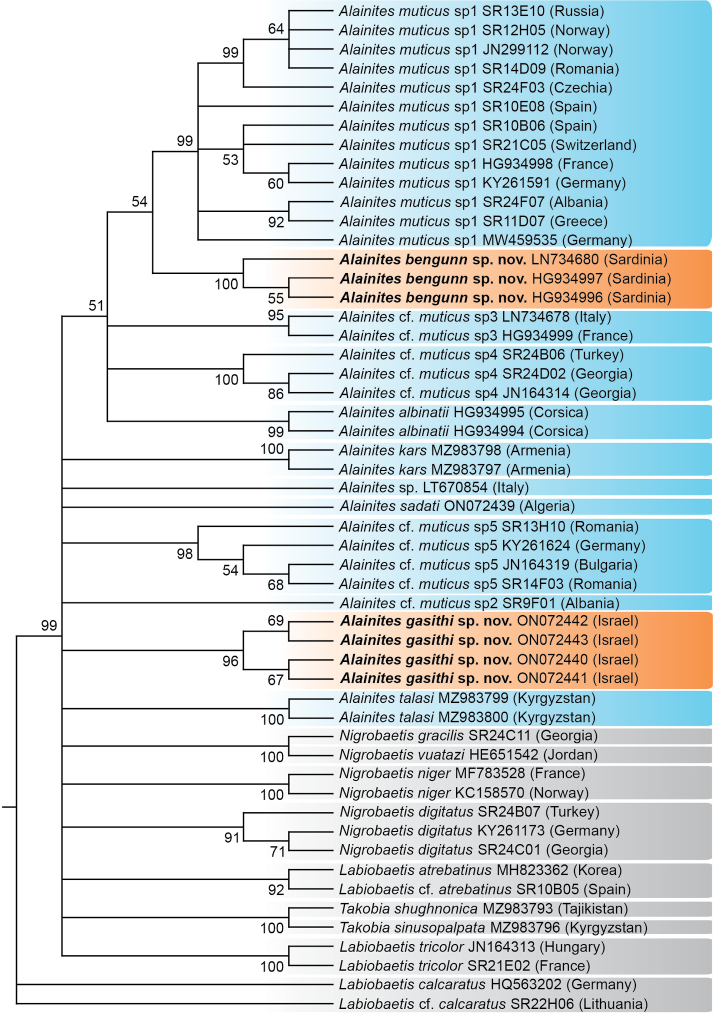
Phylogenetic reconstruction of *Alainites* species and allied taxa, based on maximum likelihood analysis of sequences of the mitochondrial COI gene. The reconstruction includes representatives of the two new *Alainites* species (displayed with orange background), all other sequenced *Alainites* species (blue), and representative species of *Nigrobaetis* and *Takobia* (grey). For full details of selected samples see Suppl. material 1. ML bootstrap values higher than 50% are indicated next to the nodes; lower than 50% are collapsed. Due to the wide polytomy in deeper nodes, branch lengths are in fact meaningless in this presentation. Taxa names are consistent with our findings, and may sometimes differ from names entered in GenBank.

*Alainitesbengunn* sp. nov. is rather similar to the Corsican *A.albinatii*. The main character to distinguish them is the presence of spines on the dorsal margin of foretibiae (ca. seven for *A.albinatii*, at least nine in *A.bengunn* sp. nov.), the shape of the spines of the distal margin of terga (slender and more pointed in *A.albinatii*) and the spines on the paraproct prolongation (the prolongation is completely covered by small spines in *A.bengunn* sp. nov., whilst only the apex is covered by spines in *A.albinatii*).

The North African species *A.sadati* and *A.oukaimeden* usually have more femoral setae and fewer tibial setae than *A.bengunn* sp. nov. (Table 1; Zrelli et al. 2012). *Alainitessadati* also exhibits more elongated labrum and apical segment of the labial palp compared to *A.bengunn* sp. nov. Furthermore, the length of maxillary palp does not exceed the apex of galealacinia in *A.sadati*, contrary to *A.bengunn* sp. nov. *Alainitesoukaimeden* possesses a different arrangement of paraproct compared to *A.bengunn* sp. nov.: the prolongation in *A.oukaimeden* is broader, and marginal spines outer to prolongation are smaller and more numerous (Thomas et al. 1992: fig. 6).

Nigrobaetis (Takobia) katerynae from Georgia represents a species not formally assigned in *Alainites*, although complying with the definition of this genus used in the present study. Until a more extensive phylogenetic analysis is done, we refrain from introducing new combinations, therefore we treat *N.katerynae* under the name originally proposed by Martynov and Godunko (2017). *Nigrobaetiskaterynae* can be distinguished from *A.bengunn* sp. nov. by multiple characters: a different structure of the area between incisors and mola of mandibles (no serration on left mandible and row of setae on right one in *N.katerynae*, whereas the space between prostheca and mola is serrated in both mandibles in *A.bengunn* sp. nov.); more elongated apical segment of labial palp in *N.katerynae*; and narrower spines on posterior margin of terga in *A.bengunn* sp. nov.

*Alainitesgasithi* sp. nov. is more distinct amongst the West Palaearctic species, mainly due to the arrangement of spines on the paraproct prolongation, i.e., only along the lateral margin. The only two other species with similar spines arrangement are easily distinguished: *A.muticus* has seven pairs of gills, and *A.kars* has no serration between prostheca and mola, plus it has many more dorsal setae on its forefemora. Moreover, at least some populations of *A.kars* possess spines on all the surface of the paraproct prolongation (Martynov and Godunko 2017: figs 45, 46). *Alainitesgasithi* sp. nov. may have the lowest number of dorsal setae on forefemora and foretibiae compared to all other West Palaearctic species, but this character should be treated with caution since it may vary to some extent and can overlap with numbers exhibited by other species.

### ﻿Phylogenetic reconstruction

The ML analysis (Fig. 7) supports the monophyly of the two new species, *A.bengunn* sp. nov. and *A.gasithi* sp. nov. No further information can be gained regarding the relationships amongst close genera, since COI is a useful barcode segment for species delineation and identification, but not informative for deeper nodes. We therefore recommend on a more thorough investigation regarding the systematics of *Alainites*, *Nigrobaetis*, and *Takobia*, which should rely on morphological or wider genetic data. An analysis of the genetic distances amongst and between populations also supports the monophyly of the newly described species (Suppl. material 2). Intraspecific variation of the COI distances never exceeds 0.7%, as expected from populations that belong to the same species. For both species, the known populations are geographically restricted, and we expect higher differences only if additional, further populations are discovered. On the other hand, each of the sequences of the new species is at least 19% different from any other *Alainites*, *Nigrobaetis*, or *Takobia* species that we have analysed, a pattern that leaves no doubt regarding their specific independence. High variation amongst *A.muticus* samples is evident, and has already been noticed by Sroka (2012) and Sroka et al. (2021). As detailed therein, these are probably due to misidentified specimens that were contributed to GenBank, and, perhaps, it is another clue for *A.muticus* being a complex of cryptic species. The latter explanation is of high importance for the resolution of *Alainites* systematics.

### ﻿Distribution of *Alainites* species

Both *A.bengunn* sp. nov. and *A.gasithi* sp. nov. are known from very restricted ranges and are endemic to Sardinia and to Israel, respectively. It is possible that further research will reveal *A.gasithi* sp. nov. populations in Jordan, Syria, or Lebanon, although no suspicious species have been reported from there to date (e.g., Koch 1988, Gattolliat et al. 2012, Alhejoj et al. 2020). Being so geographically restricted, the two species follow the knowledge of the distribution of other West Palaearctic *Alainites* spp.: most of them are known from very limited areas, and almost no sympatry is recorded, i.e., two *Alainites* species are almost never found together.

In conclusion, identification of nymphs of *Alainites* based solely on morphology may be remarkably confusing. However, an integrative approach, including also molecular evidence and distribution, often allows an accurate species delimitation and a reliable identification. Similar approach should be applied to solve the potential presence of cryptic species within *Alainitesmuticus* s. l. and the assignment of populations from Sicily (Tenchini et al. 2018) and North Morocco (Khadri et al. 2017; Mabrouki et al. 2019).

## Supplementary Material

XML Treatment for Alainites bengunn

XML Treatment for Alainites gasithi

## Supplementary materials

Supplementary material 1 Table S1 Data type docx file. Explanation note Taxa used for genetic distance analysis (mitochondrial COI sequences) with GenBank accession numbers or FREDIE reference codes (novel sequences are highlighted in bold font). https://binary.pensoft.net/file/734636This dataset is made available under the Open Database License (http://opendatacommons.org/licenses/odbl/1.0/). The Open Database License (ODbL) is a license agreement intended to allow users to freely share, modify, and use this Dataset while maintaining this same freedom for others, provided that the original source and author(s) are credited.Zohar Yanai, Pavel Sroka, Jean-Luc Gattolliat

Supplementary material 2 Table S2 Data type docx file. Explanation note Kimura 2 parameter distance amongst sequences of the mitochondrial COI gene of selected Alainites, Takobia, and Nigrobaetis species. https://binary.pensoft.net/file/734637This dataset is made available under the Open Database License (http://opendatacommons.org/licenses/odbl/1.0/). The Open Database License (ODbL) is a license agreement intended to allow users to freely share, modify, and use this Dataset while maintaining this same freedom for others, provided that the original source and author(s) are credited.Zohar Yanai, Pavel Sroka, Jean-Luc Gattolliat
