# Supplementary material for: ﻿Two new species of Alainites (Ephemeroptera, Baetidae) from the Mediterranean biodiversity hotspot
Source: Zookeys. 2022 Aug 24;1118:73–95. doi: 10.3897/zookeys.1118.84643 (PMC9848644; doi:10.3897/zookeys.1118.84643)
Supplement: Supplementary material 1 — Table S1 [file zookeys-1118-073_article-84643__-s001.docx]

**Supplementary material 1.**

Table S1. Taxa used for genetic distance analysis (mitochondrial COI sequences) with GenBank accession numbers or FREDIE reference codes (novel sequences are highlighted in bold font). Taxa names are consistent with our findings, and may sometimes differ from names entered in GenBank.

|  | Source of samples | FREDIE reference | GenBank accession number |
| --- | --- | --- | --- |
| *Alainites bengunn* sp. nov. | Sardinia (Italy) |  | HG934996, HG934997, LN734680 |
| *Alainites gasithi* sp. nov. | Israel |  | **ON072440**, **ON072441**, **ON072442**, **ON072443** |
| *Alainites* cf. *muticus* sp1 | Albania, Czechia, France, Germany, Greece, Norway, Romania, Russia, Spain, Switzerland | SR12H05, SR14D09, SR13E10, SR24F03, SR10B06, SR21C05, SR10E08, SR11D07, SR24F07 | JN299112, HG934998, KY261591, MW459535 |
| *Alainites* cf. *muticus* sp2 | Albania | SR9F01 |  |
| *Alainites* cf. *muticus* sp3 | France, Italy |  | LN734678, HG934999 |
| *Alainites* cf. *muticus* sp4 | Georgia, Turkey | SR24B06, SR24D02 | JN164314 |
| *Alainites* cf. *muticus* sp5 | Bulgaria, Germany, Romania | SR14F03, SR13H10 | JN164319, KY261624 |
| *Alainites albinatii* | Corsica (France) |  | HG934994, HG934995 |
| *Alainites sadati* | Algeria |  | **ON072439** |
| *Alainites kars* | Armenia |  | MZ983797, MZ983798 |
| *Alainites talasi* | Kyrgyzstan |  | MZ983799, MZ983800 |
| *Nigrobaetis niger* | Norway |  | KC158570, MF783528 |
| *Nigrobaetis gracilis* | Georgia | GE006 |  |
| *Nigrobaetis vuatazi* | Jordan |  | HE651642 |
| *Nigrobaetis* cf. *digitatus* | Georgia, Germany, Turkey | TR008, GE007 | KY261173 |
| *Labiobaetis atrebatinus* | South Korea |  | MH823362 |
| *Labiobaetis* cf. *atrebatinus* | Spain | ES012 |  |
| *Labiobaetis tricolor* | France | FR031 | JN164313 |
| *Labiobaetis* cf. *calcaratus* | Germany, Lithuania | LT014 | HQ563202 |
| *Takobia* sp. | Sicily (Italy) |  | LT670854 |
| *Takobia shughnonica* | Tajikistan |  | MZ983793 |
| *Takobia sinusoplapata* | Kyrgyzstan |  | MZ983796 |
