# Supplementary material for: ﻿Two new species of Alainites (Ephemeroptera, Baetidae) from the Mediterranean biodiversity hotspot
Source: Zookeys. 2022 Aug 24;1118:73–95. doi: 10.3897/zookeys.1118.84643 (PMC9848644; doi:10.3897/zookeys.1118.84643)
Supplement: Supplementary material 2 — Table S2 [file zookeys-1118-073_article-84643__-s002.docx]

Supplementary material 2.

Table S2. Kimura 2 parameter distance amongst sequences of the mitochondrial COI gene of selected *Alainites*, *Takobia*, and *Nigrobaetis* species (presenting mean and min–max distance for each group with > 1 individual; for number of samples per taxon see Supplementary material 1). Taxa names correspond to the sequences detailed in Supp. material 1, using the following abbreviations: Ab = *Alainites bengunn* sp. nov., Ag = *Alainites gasithi* sp. nov., A1 = *Alainites* cf. *muticus* sp1, A2 = *Alainites* cf. *muticus* sp2, A3 = *Alainites* cf. *muticus* sp3, A4 = *Alainites* cf. *muticus* sp4, A5 = *Alainites* cf. *muticus* sp5, Aa = *Alainites albinatii*, As = *Alainites sadati*, Ak = *Alainites kars*, At = *Alainites talasi*, T = *Takobia* spp., N = *Nigrobaetis* spp., L = *Labiobaetis* spp.

| Ab | 0.5%  0.1–0.7% |  |  |  |  |  |  |  |  |  |  |  |  |  |
| --- | --- | --- | --- | --- | --- | --- | --- | --- | --- | --- | --- | --- | --- | --- |
| Ag | 22.4%  22.1–22.7% | 0.1%  0.0–0.1% |  |  |  |  |  |  |  |  |  |  |  |  |
| A1 | 20.3%  19.6–21.0% | 22.7%  22.2–23.1% | 1.2%  0.0–2.3% |  |  |  |  |  |  |  |  |  |  |  |
| A2 | 26.6%  26.3–26.7% | 23.1%  23.0–23.2% | 21.8%  21.2–22.3% | – |  |  |  |  |  |  |  |  |  |  |
| A3 | 21.0%  20.6–21.5% | 24.8%  24.4–25.2% | 17.9%  16.8–18.9% | 23.5%  23.3–23.7% | 2.1% |  |  |  |  |  |  |  |  |  |
| A4 | 20.0%  19.1–21.1% | 24.5%  24.3–24.9% | 18.1%  16.6–19.1% | 23.3%  22.7–24.1% | 16.9%  16.3–17.8% | 0.1%  0.1–0.2% |  |  |  |  |  |  |  |  |
| A5 | 27.2%  26.4–28.4% | 22.5%  21.4–25.2% | 22.9%  21.6–23.9% | 23.8%  22.9–26.7% | 23.5%  22.9–24.2% | 23.5%  22.7–24.4% | 0.8%  0.3–1.3% |  |  |  |  |  |  |  |
| Aa | 19.6%  19.2–19.8% | 23.0%  22.6–23.3% | 16.8%  16.5–17.3% | 20.1%  20.3–21.0% | 17.5%  17.4–17.7% | 19.8%  18.8–21.1% | 20.7%  19.7–22.4% | 1.5% |  |  |  |  |  |  |
| As | 24.7%  24.3–25.3% | 23.9%  23.8–24.1% | 23.7%  23.5–24.4% | 25.0% | 23.8%  23.2–24.3% | 26.0%  25.7–26.5% | 19.9%  19.3–20.4% | 23.0%  22.9–23.2% | – |  |  |  |  |  |
| Ak | 24.7%  24.3–25.0% | 19.4%  19.2–19.6% | 22.1%  21.4–22.5% | 24.2%  24.1–24.3% | 23.3%  22.1–24.4% | 25.9%  24.8–27.6% | 20.0%  19.1–21.6% | 20.1%  19.9–20.3% | 23.5%  23.4–23.6% | 0.1% |  |  |  |  |
| At | 27.1%  27.1–27.1% | 24.0%  23.9–24.1% | 27.5%  26.6–28.1% | 26.4% | 24.8%  24.3–25.4% | 28.0%  27.9–28.1% | 25.1%  24.6–25.8% | 27.4%  27.1–27.6% | 25.0%  25.0–25.0% | 24.9%  24.8–25.0% | 0.0% |  |  |  |
| T | 27.2%  26.2–28.0% | 23.6%  22.9–24.3% | 25.6%  24.7–26.6% | 26.7%  25.7–27.6% | 24.6%  24.2–25.1% | 27.1%  24.2–30.3% | 25.9%  23.1–29.5% | 22.2%  21.9–22.8% | 26.6%  26.2–27.0% | 23.0%  22.7–23.3% | 24.2%  23.0–25.4% | 13.5% |  |  |
| N | 27.4%  20.6–30.0% | 23.3%  20.4–25.5% | 25.7%  22.3–27.8% | 27.6%  25.2–29.9% | 25.5%  21.4–29.2% | 26.7%  23.8–30.2% | 23.4%  21.3–25.9% | 22.8%  19.9–25.5% | 23.6%  18.9–27.3% | 22.2%  19.1–24.1% | 23.5%  21.9–24.7% | 23.6%  19.9–26.1% | 19.7%  1.9–23.9% |  |
| L | 25.0%  23.2–26.9% | 22.7%  21.3–23.8% | 24.2%  22.4–25.8% | 26.8%  26.0–27.9% | 24.4%  22.4–27.6% | 23.6%  22.4–25.2% | 24.2%  21.6–27.2% | 21.7%  19.6–25.9% | 23.5%  21.6–26.0% | 22.3%  19.6–25.4% | 23.7%  22.7–24.8% | 22.3%  20.5–25.7% | 21.6%  17.6–24.3% | 15.9%  0.4–21.0% |
|  | Ab | Ag | A1 | A2 | A3 | A4 | A5 | Aa | As | Ak | At | T | N | L |
